# Normal pressure hydrocephalus – potentially treatable dementia and window for living human brain

**DOI:** 10.1002/alz70856_097566

**Published:** 2025-12-24

**Authors:** Ville Leinonen

**Affiliations:** ^1^ Kuopio University Hospital, Kuopio, Finland

## Abstract

**Background:**

Normal pressure hydrocephalus (NPH) is a memory and movement disorder covering up to 5% of all dementia. Familial aggregation in up to 20% of NPH is a strong indicator of notable genetic component in the pathobiological process. Number of genetic variants and disease associated loci have been identified in NPH. These findings seem to be independent from neurodegenerative diseases but are linked with blood brain borders, brain ventricle volume and CSF biomarker levels. The most important differential diagnosis and comorbidity is Alzheimer's disease (AD).

**Method:**

Kuopio NPH cohort include over 1200 patients with possible or probable NPH, frontal cortical brain biopsy and life‐long follow‐up since 1993 till date. Biopsies have systematically immunohistochemically stained against amyloid β (Aβ) and tau. Currently, the fresh biopsy is shortly after extraction sliced also for immediate electrophysiology and frozen for transcriptomic analysis.

**Result:**

Half of the patients have various degree of Aβ pathology, around 10% have both Aβ and tau pathology and up to 5% have tau pathology without Aβ. CSF biomarkers of AD (Aβ1‐42, *p*‐Tau181 and T‐Tau) are generally decreased in NPH but correlate well with brain biopsy AD‐related pathologies. Since Aβ pathology occur early in frontal cortex, the biopsy is sensitive for Aβ pathology and subsequent clinical AD. On the other hand, the biopsy is not so sensitive for tau since cortical tau occur later but when positive indicate already an advanced pathology (Braak stage V‐VI).

**Conclusion:**

Amyloid plaques even in tiny frontal (or parietal) brain biopsy taken during shunt surgery can indicate high risk of comorbid clinical AD and together with tau, neuropathologically verified biological AD or rarely seen tau pathology alone, a primary tauopathy like frontotemporal dementia (FTD). Furthermore, obtainment of living human brain samples, are valuable for omics techniques and imaging at single cell level to uncover individual cell level disease mechanisms related with NPH and AD as well as other neurodegenerative diseases seen in NPH patients and hampering their prognosis.

